# Impact of Age and Sex on Extra‐Brain, Off‐Target Binding in Tau PET Scans (18F‐FTP)

**DOI:** 10.1002/alz70856_107719

**Published:** 2026-01-11

**Authors:** Sujala Ghatamaneni, Daneil Kim, Marin E Nycklemoe, Yurim Claire Choi, Christopher Apgar, Mahathi Kandimalla, David N Jacobson, Tiffany Kung, Vamika Sharma, Seokbeen Lim, Haakon Hol, Emily S. Lundt, Sabrina M. Albertson, Christopher G Schwarz, Jeffrey L. Gunter, Ronald Petersen, Clifford R. Jack, Val J Lowe, Hoon‐Ki Min

**Affiliations:** ^1^ Mayo Clinic, Rochester, MN, USA; ^2^ Sørlandet Hospital HF, Arendal, Arendal, Norway; ^3^ Department of Radiology, Mayo Clinic, Rochester, MN, USA

## Abstract

**Background:**

The tau PET imaging with 18F‐flortacipir (18F‐FTP) has focused interest in accurately tracking longitudinal tau changes in Alzheimer's disease [1]. Thus, minimizing variability is critical. Off‐target signal has the potential to complicate interpretation of tau‐PET imaging due to potential spillover or bleed‐in effects, with increased effects seen in later stages of Alzheimer's due to cerebral atrophy and proximity of off‐target areas to relevant cortical areas[2]. Previously, we demonstrated the effect of uptake period activity on 18F‐FTP signal. With increasing recent interest in demographic factors that may affect off‐target binding, we investigated the effect of age in extra‐cerebral off‐target signal in 18F‐FTP PET imaging.

**Method:**

Standard 18F‐FTP PET protocol was performed in 330 participants. Two independent readers measured tau uptake in predetermined off‐target regions, including extraocular muscle, meninges, and occipital bone among others. Linear regression models assessed the relationship between participants’ age, sex, and tau uptake values (SUV and SUVr) (SUV = (Tissue radioactivity concentration / Injected dose) / Body weight & SUVr = SUV of target region/ SUV of reference region.)

**Result:**

Sex had a significant effect on off‐target signal in most brain regions, with variable positive or negative correlation based on area. Notably, SUV in the eyeball‐retina, occipital bone, upper skull meninges all showed significant positive correlation with sex, with ‘male’ status generally associated with lower SUV. The greatest effect of sex was observed in superior skull meninges, occipital bone, and internal carotid artery (*p* <0.001). Age showed minimally affected SUV and SUVr in off‐target regions. SUVr values show a significant correlation with SUV values of upper skull meninges in sex. Cognitive status did not significantly affect off‐target SUV.

**Conclusion:**

We observed that sex significantly affected extra‐brain off‐target signal in 18F‐FTP PET imaging, while age has minimal effect, adding to previous evidence of off‐target binding within brain regions. Further evaluation is necessary to understand the age‐related effects on off‐target regions. As a result, the variability introduced by sex may pose challenges to studying tau accumulation.

